# Psychological distress does not predict decisional regret in patients undergoing spinal reconstruction for adult spinal deformity

**DOI:** 10.1186/s12891-024-08126-1

**Published:** 2024-12-03

**Authors:** Jesse Shen, Philip Louie, Takeshi Fujii, Caroline E. Drolet, Aiyush Bansal, Venu Nemani, Jean-Christophe Leveque, Rajiv Sethi

**Affiliations:** 1 Department of Neurosurgery, Center for Neuroscience and Spine, Virginia Mason Medical Center, Seattle, WA USA; 2 Faculty of Medicine, Department of Surgery, CHUM, University of Montreal, Montreal, QC Canada

**Keywords:** Psychological regret, Decisional regret, Adult spinal deformity, SRS-22

## Abstract

**Purpose:**

The study aimed to assess the link between preoperative psychological distress and postoperative decisional regret in adult spinal deformity (ASD) surgery patients. We hypothesized that greater pre-surgery distress would correlate with higher post-surgery regret. This evaluation was based on a retrospective case series from an institution with standardized surgical guidelines for ASD.

**Methods:**

This IRB-approved retrospective study analyzed our institution’s ASD database from 2014 to 2020. Eligible patients had a minimum two-year post-op follow-up and preoperative psychological distress assessment. Patients were grouped based on psychological distress levels: green, yellow, and yellow minus. Regret post-surgery was assessed using the Decision Regret Scale and SRS-22 Question 22. Logistic regression evaluated the impact of distress levels on regret, controlling for age and sex.

**Results:**

Out of 167 eligible patients, 112 responded and were analyzed. No significant demographic differences were observed between responders and non-responders. Using the Decision Regret Scale, 41% expressed no regret, while 63% expressed no regret with the SRS-22 questionnaire’s Single-Item scale. Only the yellow minus group showed significant regret difference based on osteotomy, with non-recipients more likely to express regret.

**Conclusion:**

This study found no significant link between psychological distress and post-operative regret in adult spinal deformity surgery after a minimum 2-year follow-up. Although nearly 60% exhibited some post-surgery regret, predicting regret based on psychological burden or demographics remains challenging. Further research is essential to identify factors contributing to post-operative regret in spinal deformity surgery patients.

## Introduction

Symptomatic adult spinal deformity (ASD) is a debilitating condition that is comparable in burden to medical conditions such as diabetes and heart failure with a prevalence as high as 68% amongst older adults [1, 2]. The burden of ASD on health systems will be increasingly significant as the amount of surgical pathology and utilization is likely to rise due to an aging population [3, 4]. Recent studies have demonstrated the effectiveness of surgical management of adult spinal deformity [5, 6]. Nielsen et al. demonstrated that almost 70% of patients managed surgically had significant improvement in patient reported outcomes however, this result potentially leaves 30% of surgical patients with a suboptimal outcome [5]. Moreover, as recent studies have reported a 20% rate of unexpected readmission and 45% overall complication rate in ASD surgery, the role for shared decision-making becomes extremely critical for both patients and clinicians weighing the potential benefits and risks of surgery [7].

This decision making is a complex interplay of several factors such as personal values, preferences, desired outcomes and risk tolerance [8]. Patients who are well-informed about the risks and benefits of a procedure are likely to have better outcomes and less regret about their decision [9]. However, Sikora et al. demonstrated that only 17% of patients with spinal deformity had a history devoid of mental illness and also had a reasonable expectation of surgery, suggesting that this decision-making process may be more complicated in this population [10]. Psychological comorbidity becomes an important factor in decision making for spinal deformity surgery. Several reports have shown that comorbid conditions such as anxiety or depression have an impact on outcomes after surgery [11, 12].

A recent publication demonstrated that 20% of patients had medium to high levels of regret following adult spinal deformity surgery [13]. An association was noted between preoperative depression and decisional regret, suggesting that psychological comorbidity may have an impact on decisional regret. Tools to evaluate this psychological burden will be important in creating an optimal surgical shared decision-making process.

The objective of this study was to evaluate the relationship between a patient’s preoperative psychological distress and postoperative decisional regret following adult spinal deformity surgery. We hypothesized that patients with high psychological distress would experience higher decisional regret post-operatively as assessed by a single-center retrospective consecutive case series of patients surgically treated for ASD in an institution with published standardized surgical guidelines [14].

## Methods

### Patient inclusion

This study was approved by the institutional review board of our medical center. Our institutional ASD database is a prospectively-maintained database of all spinal deformity cases surgically treated at our institution. We characterize adult spinal deformity as scoliosis, kyphosis, or flat-back or any revision case that requires at least 6 levels of fusion. This is in line with the initial Seattle Spine Team approach [14]. Patients who had surgery between January 1st _,_ 2014 to May 1st, 2020 were eligible for inclusion in this study. We included any patients with a minimum of two-year post-op follow-up and a preoperative psychological distress assessment using a previously published methodology and described below [10]. The current study was approved by our institutional review board (IRB21-055) by Benaroya Research Institute. A full waiver of HIPPA and signed consent was approved for this study. Eligible patients were contacted by phone and verbal consent was obtained prior to any further study discussion. A patient was contacted a minimum of 5 times before they were deemed unreachable and excluded from analysis. Each patient who decided to enroll in the study had 5 reminders at 5 different time intervals to complete the study survey; if they failed to complete the study survey after these reminders they were excluded from analysis.

Patients were excluded if they were treated surgically in an urgent or non-elective fashion, as were those whose primary diagnosis leading to surgical treatment was tumor, infection or acute trauma.

### Psychological distress assessment

The psychological distress assessment is a comprehensive evaluation tool performed by a clinic psychologist which uses several validated cognitive tools as well as measures to assesses a patient’s expectation of surgery, mental health disorders as well as substance abuse history. For the data analysis, the final result of this psychological distress assessment is stratified with color-code grading in increasing order of severity of psychological burden: green (including green minus), yellow, yellow minus (including orange) [10]. The analysis was performed with these three groups based on preoperative assessment as there are far fewer patients in the extremes of this classification such as green, orange or red.

### Regret evaluation tools

Two tools were utilized to assess decisional regret. The decisional regret scale is a simple and validated 5 item scale that was developed to evaluate decisional regret in medical decision making [15]. Question 22 of the SRS-22 questionnaire was also utilized (“Would you have the same management again if you had the same condition?”) as the SRS-22 is validated tool to assess patient reported outcomes in ASD and this question covers the theme of decisional regret and patient satisfaction or dissatisfaction [16]. Patients completed these questionnaires online through RedCap via our institution’s account. Patients were offered an opportunity to complete the survey in person or through telephone communication if online access was not possible. The regret results were interpreted separately as these are two different scales.

### Demographic and surgical variables

Demographic variables collected included age, sex and comorbidity burden as measured by ASA class. Intraoperative and postoperative variables including numbers of levels fused and instrumented levels were obtained through our complex spine database.

### Statistical analysis

Composite scores for the Decision Regret Scale were calculated according to the instructions from O’Connor [15]. The scale demonstrated good reliability with a Cronbach’s alpha = 0.93. Responses for both the regret scale composite and the single-item regret measure from the SRS-22 were extremely leptokurtic and positively skewed. To account for this non-normality, we created bivariate scores such that 0 indicates no regret (a composite score of 1 or responding 1 on the single item measure) and 1 indicates some amount of regret.

We used logistic regression for our main hypothesis tests. For all main hypothesis tests, we controlled for age at surgery and patient sex. We examined the effect of color group, reoperation, osteotomy, and time since surgery on each of the regret measures. The color group was dummy-coded such that green was the comparison group. We also examined interactions that could potentially influence regret such as between color group and reoperation, osteotomy, and time since surgery. Any significant or marginal interactions were deconstructed by examining the slopes at each color level. Statistical significance was defined as *p* < .05 and 95% confidence intervals (CIs) with the odds ratio intervals containing 1 deemed non-significant.

## Results

### Demographics

A total of 112 patients (67%) who were eligible (167 patients) and responded to the surveys were analyzed (Table 1). Both previously operated (*n* = 77) and index deformities (*n* = 35) were included. The average levels fused was 10.2 ± 3.7. 104 patients were instrumented to the pelvis. There were no significant demographic differences between those who responded and those who did not. There was no significant difference amongst the psychological distress groups based on age, sex and ASA (Table 2).

**Table 1:** Patient demographics with comparison between survey responders and non-responders

		Total				Survey Answered				Survey Refused			*p*
		*N*	*M*	*SD*		*N*	*M*	*SD*		*N*	*M*	*SD*	
Age		167	62.4	11.7		112	62.3	12.7		55	62.7	9.4	0.82
BMI		166	29.7	6.8		112	29.5	6.8		54	30.1	6.6	0.57
		*N*	%			*N*	%			*N*	%		
Sex	Female	107	64.1%			74	66.1%			33	60%		0.55
	Male	60	35.9%			38	33.9%			22	40%		
ASA	I	1	< 1%			1	< 1%			0	0%		0.11
	II	73	44.2%			44	39.6%			29	53.7%		
	III	91	55.2%			66	59.5%			25	46.3%		

Note. All *p*-values are for comparisons between Survey Answered and Survey Refused groups. Unpaired t-tests were used for all comparisons of age and BMI. Wilcoxon-ranked sum test was used for all comparisons of ASA class. Chi-squared was used to compare proportions of sex between survey groups

**Table 2:** Characteristics of patients classified by distress stratification group

	Green (*n* = 29)	Yellow (*n* = 57)	Yellow Minus (*n* = 26)	*p*
Age	60.36 (16.23)	64.58 (11.23)	61.38 (11.15)	.29_a_
Sex				.80_b_
Female	20 (17.86%)	36 (32.14%)	18 (16.07%)	
Male	9 (8.04%)	21 (18.75%)	8 (7.14%)	
ASA				.20_c_
I	1 (0.89%)	1 (0.89%)	0 (0.00%)	
II	18 (16.07%)	39 (34.82%)	12 (10.71%)	
III	10 (8.93%)	17 (15.18%)	14 (12.50%)	

Note. Standard deviations in parentheses for continuous variables and percentages for discreet variables

_a_ = ANOVA

_b_ = Chi-squared test

_c_ = Fisher’s exact test

### Descriptive data

Table 3 summarizes the results of both the Decision Regret Scale and the Single-Item Regret Measure of the SRS-22 questionnaire. 41% of patients were “not regretful” based on the Decision Regret Scale while 63% of patients were “not regretful” based on the Single-Item scale of the SRS-22 questionnaire. Patients were classified as “not regretful” if all answers were devoid of any regret such as “Definitely Yes” for the SRS-22 questionnaire and a score of 0 for the Decision Regret Scale.

**Table 3:** Summary of survey responses for the decision regret scale and SRS question 22

		*n*	not regretful	regretful	% not regretful	% regretful	mean	sd	min	max	ANOVA F	ANOVA *p*	Chi-squared	Chi-squared *p*
	green	29	13	16	44.83%	51.17%	1.49	0.66	1	3.25	1.148	0.321	0.25445	0.8805
Decision Regret Scale	yellow	57	23	34	40.35%	59.65%	1.74	0.93	1	5				
	yellow minus	26	10	16	38.46%	61.54%	1.85	1.14	1	5				
	**TOTAL**	112	46	66	41.07%	58.93%	1.7	0.93	1	5				
	green	29	20	9	68.97%	31.03%	1.44	0.78	1	4	1.961	0.146	1.4179	0.4922
SRS question 22	yellow	56	36	20	64.29%	35.71%	1.63	1.04	1	5				
	yellow minus	26	14	12	53.85%	46.15%	2	1.33	1	5				
	**TOTAL**	111	70	41	63.06%	36.94%	1.67	1.06	1	5				

### Decision regret scale

There were no significant main effects for any of the predictors (*p*s > 0.09). No other interaction terms approached significance including the length of time since surgery.

### Single-item regret measure

There were no significant main effects for any of the predictors including the UIV selection level (*p*s > 0.05). For those in the yellow minus group, those who did not receive an osteotomy were significantly more likely to regret their decision than those who did receive an osteotomy, *b* = -2.00, *p* = .04, odds ratio = 0.13, 95% CI of odds ratio [0.02, 0.94]. Slopes in the other color groups did not significantly differ from 0, *p*s > 0.07. No other interaction terms approached significance.

## Discussion

The results of this study reject the hypothesis that patients with higher levels of psychological comorbidity, such as a color grade of yellow or worse, have a significantly higher chance of postoperative decisional regret than those with minimal or absent psychological burden. The results showed that postoperative decisional regret was not significantly associated with patient psychological distress, reoperations and time since surgery, nor was it associated with any demographic variables or measured intraoperative and postoperative conditions. These findings contribute to the ongoing efforts to understand and characterize post-operative patient decisional regret after spinal deformity surgery.

Our initial hypothesis was formed based on several observations that the presence of preoperative mental health conditions was associated with increased decisional regret or poorer patient reported outcomes [13, 17]. The psychological distress stratification utilized in this study is a comprehensive assessment of psychological comorbidity, and we hypothesized therefore that higher distress would be associated with higher degree of regret. The current finding suggest that while the presence of a specific mental health condition of depression may lead to increased risk of regret, specific factors associated with the regret are poorly understood in patients with higher levels of psychological distress versus those with a specific mental health condition [18].

It is possible that our extensive pre-operative counseling and teaching process may have minimized the effect of psychological distress on regret. Previous reports have shown that as little as 17% of patients had realistic expectations of surgery [10]. Although all patients underwent the same pathway of care, given that psychological distress evaluations incorporate an assessment of patient expectations of surgery, patients with more unrealistic expectations at baseline may gain the most from this process and therefore have a decreased chance of experiencing postoperative regret [19].

We analyzed the results of the regret questionnaire as a continuous variable without categorizing patients based on levels of regret in an effort to minimize any threshold bias. Although a previous study utilized a discrete scale from 0 to 100 to categorize regret, the threshold was arbitrarily defined as 40 within this study, potentially introducing bias towards or against a particular endpoint [13]. In our cohort, 41% of patients demonstrated “no regret” based on the Decision Regret Scale and 63% of patients showed “no regret” based on the SRS-22 scale. It may be the case that patients have some dissatisfaction with surgery or a regret regarding particular outcomes that is not able to be determined by these measures or within the confines of our collected database variables.

There are several limitations to this study. First, this study was limited by a lack of complete patient reported outcome scores. Only 50% of eligible patients had patient reported outcome scores available at the time of the study and this measure was therefore not included in the analysis. The impact of patient reported outcomes including the presence or lack of residual pain should be further investigated in addition to another study evaluating residual malalignment with regretfulness. Another limitation is the retrospective single center design of this study, although our center has a standardized process of care both pre-operative and post-operatively that should have mitigated potential treatment bias. Since only patients with a minimum of 2-year follow-up were included for analysis and since our regret measures were only gathered after this two-year timepoint, there may be an early regret about the decision for surgery that is not captured within this analysis. Furthermore, there may be a selection bias as only 66.7% of eligible patients responded to the surveys. Although this response, is higher than that in previous reports, it is possible that patients with higher degrees of regret were less inclined to be included in our analysis, thereby skewing the results [13].

## Conclusion

We demonstrate here that psychological distress stratification was unable to predict post-operative decisional regret at a minimum of 2-years following adult spinal deformity surgery. Although we show that almost 60% of patients may show some levels of regret after surgery, it may be difficult to preoperatively identify those patients who are likely be considered regretful following surgery based on their demographics or a measure of psychological burden. Understanding the factors that may contribute to this feeling of regret may be critical as another measure of patient outcome in spinal deformity surgery. Further work will be required to examine the nature and source of regret in patients undergoing spinal deformity surgery.

## Data Availability

The datasets used and/or analysed during the current study available from the corresponding author on reasonable request.
